# Risk Profiles and Outcomes of Uterine Rupture: A Retrospective and Comparative Single-Center Study of Complete and Partial Ruptures

**DOI:** 10.3390/jcm14144987

**Published:** 2025-07-15

**Authors:** Sunhwa Baek, Valeria Froese, Bernd Morgenstern

**Affiliations:** Department of Obstetrics and Gynecology, Medical Faculty and University Clinic of Cologne, University of Cologne, 50937 Cologne, Germanybernd.morgenstern@uk-koeln.de (B.M.)

**Keywords:** high-risk uterine rupture, complete uterine rupture, partial uterine rupture, de novo uterine rupture, postpartum hemorrhage, vaginal birth after cesarean, antepartum abdominal pain after cesarean

## Abstract

**Background:** Uterine rupture is a rare but severe obstetric complication with significant maternal and neonatal consequences. While partial uterine ruptures (PURs) are generally associated with less severe outcomes, complete uterine ruptures (CURs) carry a higher risk of serious impact on both mother and child. The present study aimed to evaluate outcomes and identify risk factors for each type of rupture, and also to define high- and low-risk uterine ruptures based on clinical outcomes. **Methods:** A retrospective analysis of 112 uterine rupture cases, including 29 CURs and 83 PURs, was conducted at the Women’s Hospital of the University of Cologne from October 2010 to January 2021. **Results:** Maternal outcomes revealed that CUR was associated with higher risks of prolonged hospitalization (*p* = 0.003), postpartum hemorrhage (*p* < 0.001), maternal transfusion (*p* = 0.003), and ICU transfer (*p* = 0.004) compared to PUR. Neonatal outcomes showed a significantly higher risk of severe acidosis (*p* < 0.001), low APGAR scores (*p* < 0.001), NICU transfers (*p* = 0.004), and resuscitation needs (*p* = 0.016) in CUR cases. Factors increasing the risk of CUR included pathological CTG (OR = 1.9, 95% CI: 0.99–7.14, *p* = 0.05), abdominal pain (OR = 2.63, 95% CI: 1.10–6.25, *p* = 0.03), previous vaginal birth (OR = 7.14, 95% CI: 0.025–20, *p* < 0.001), and no uterine contractions (OR = 7, 95% CI: 1.21–40.56, *p* = 0.03). A previous cesarean section significantly increased the risk of CUR (OR = 4.94, 95% CI: 1.38–17.67, *p* = 0.014), whereas more than two cesarean sections reduced the risk (OR = 0.66, 95% CI: 0.13–3.22, *p* = 0.61). A comparison of CUR with maternal and neonatal high-risk rupture groups revealed that low gestational age and a history of previous cesarean sections were significant risk factors for neonatal high-risk rupture. **Conclusion:** Vaginal birth and abdominal pain were identified as key risk factors for CUR, which lead to severe maternal and neonatal outcomes. Recognizing these risk factors can help clinicians optimize risk stratification and decision-making, and enhance monitoring strategies to prevent adverse outcomes.

## 1. Introduction

Uterine rupture is a rare but serious complication in obstetrics, which has significant implications for both maternal and neonatal outcomes. Anatomically, it can be classified into complete rupture and partial rupture (dehiscence). A complete uterine rupture (CUR) involves the separation of all three layers of the uterine wall: the endometrium, myometrium, and perimetrium. In contrast, partial uterine rupture (PUR) is characterized by incomplete division of the uterus that spares the perimetrium and is often a prepartum undetected finding during labor in asymptomatic patients [1].

Incidence of uterine rupture varies strongly depending on factors such as previous uterine surgery, delivery modes, and healthcare access in different regions. In women who have had a previous cesarean section (C-section), the risk of a uterine rupture during a trial of labor after cesarean (TOLAC) is about 0.5%. In contrast, the risk is less than 0.02% in women who have an elective repeat C-section without labor [2]. PUR, or dehiscence of the uterus, occurs more frequently, with an estimated incidence of up to 3.8% [3]. In women with no prior uterine scar, spontaneous uterine rupture is extremely rare, occurring in about 1 in 15,000 deliveries [4,5]. The incidence of uterine rupture is higher in low-resource settings, where prolonged or obstructed labor is more common due to limited access to emergency obstetric care [6,7].

Clinical symptoms associated with uterine rupture include acute abdominal pain, abnormal cardiotocography (CTG), vaginal bleeding, unstable hemodynamics such as hypotension, impaired consciousness, tachycardia, acute absence of contractions, and dystocia [5,8].

Risk factors for uterine rupture include a previous uterine scar, excessive use of oxytocin or prostaglandins, a short inter-delivery interval, maternal age over 40, obesity, macrosomia, multiple pregnancy, abnormal placentation, the use of fundal pressure during labor, and obstructed labor [9,10,11]. In particular, the number and type of previous uterine surgeries are highly significant. The risk of uterine rupture is approximately 1% in women with a single previous cesarean delivery, compared to 3.9% in those with more than one prior cesarean delivery [1].

Globally, the rate of C-section deliveries has been steadily increasing. In Germany, the rate of C-sections doubled between 1991 and 2020 [12]. This trend has raised significant concerns about long-term consequences, which may negatively impact subsequent pregnancies, presenting a challenge in the management of labor following a C-section.

The outcomes of uterine rupture depend on its severity. Because CUR involves full-thickness separation of the uterine wall, it may lead to extrusion of the fetus or placenta into the abdominal cavity, resulting in a significant risk of maternal and fetal morbidity and mortality [9,11]. In contrast, PUR typically leads to milder consequences as an incidental finding during C-section. Thus, early detection of CUR could significantly improve maternal and neonatal outcomes.

The present study aimed to evaluate outcomes and identify risk factors specific to each rupture type. By distinguishing between CUR and PUR, we implemented a risk stratification approach that categorizes uterine ruptures based on clinical severity, which is essential for early intervention and improved patient outcomes. Furthermore, we sought to define high- and low-risk uterine ruptures based on maternal and neonatal clinical outcomes, and compare them with CUR. This distinction is particularly relevant for clinical decision-making, as it provides valuable insights for risk assessment, patient counseling, and labor management, including optimized patient selection for TOLAC and tailored intrapartum monitoring, ultimately enhancing maternal and neonatal safety.

## 2. Materials and Methods

### 2.1. Study Population

**Figure 1:**
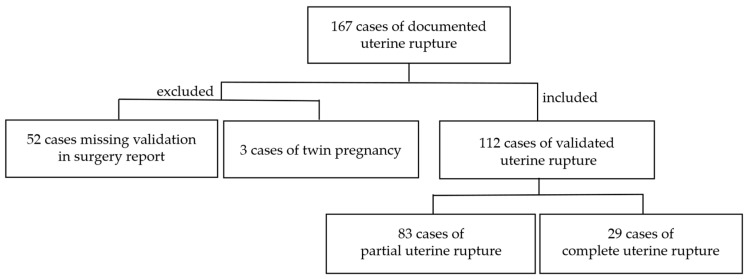
Scheme of study population.

All cases of uterine rupture that occurred between October 2010 and January 2021 at the Women’s Hospital of the University of Cologne were retrospectively analyzed. A total of 167 cases of uterine rupture were initially documented in the hospital information system ORBIS (Dedalus healthcare group, Milan, Italy, version 9); these were then cross-referenced with the corresponding operative reports. A total of 52 cases were excluded because of lack of validation. Three cases of twin pregnancy were excluded due to their inherently higher baseline risk of uterine rupture to minimize potential confounding factors related to multiple gestations. Ultimately, 112 cases of uterine rupture were confirmed based on intraoperative findings during C-section. Of these, 29 were classified as CUR, while 83 were categorized as PUR (Figure 1). As this was a retrospective study, ethical approval was not required.

### 2.2. Data Analysis

Maternal characteristics were assessed based on age at the time of delivery (≤35 years, >35 years and <40 years, ≥40 years), height (<153 cm, ≥153 cm and <180 cm, and ≥180 cm), and body mass index (BMI, <25, ≥25 and <30, and ≥30) [13]. Obstetric characteristics included gravidity and parity, as well as a history of uterine-related conditions such as C-sections, uterine surgery other than C-sections, or previous uterine ruptures. Births within short intervals and pregnancy-related risk factors, including gestational diabetes and gestational hypertension, were also analyzed [14]. Gestational ages ranged from 25 + 5 to 41 + 2 weeks; these were classified as extremely preterm birth (22 + 0–27 + 6 weeks), preterm birth (28 + 0–36 + 6 weeks), or birth at term (>37 weeks), according to the WHO definition [15].

Peripartum characteristics included labor induction, rupture of membranes before contractions, and abdominal/scar pain during the labor. The methods of induction were categorized as prostaglandin gel, catheters, oxytocin infusions for labor augmentation, or a prostaglandin insert. Prostaglandin gel, which was the most used induction method after C-section, was further analyzed according to the total amount of gel, because the maximum dose per 24 h is 3 mg [16]. Delivery modes were categorized as primary, secondary, or emergency C-section. Secondary C-section refers to a cesarean performed after the onset of labor, regardless of prior cesarean history. Another parameter was the time of delivery; specifically, whether the birth occurred during or outside of regular working hours (7:30 a.m. to 5:00 p.m.) and holidays.

Fetal characteristics such as birth weight, birth length, and head circumference were measured and categorized according to the 10th and 90th percentiles.

Maternal outcomes included postpartum hemorrhage [17], days of hospitalization, need for blood transfusion, admission to intensive care, and emergency hysterectomy. Maternal high-risk uterine rupture was defined as blood loss of more than 1000 mL after C-section which led to blood transfusion and, eventually, to transfer to the intensive care unit (ICU).

Neonatal outcomes comprised APGAR scores at 1, 5, and 10 min, pH and base excess from umbilical artery blood [18], and hemoglobin concentration. Any need for cardiopulmonary resuscitation (CPR) or admission to the neonatal intensive care unit (NICU) was also documented. Neonatal high-risk uterine rupture was defined if the child was admitted to the NICU.

### 2.3. Statistical Analysis

Nominal variables were analyzed using a chi-square test or Fisher’s exact test according to sample size. For ordinal variables, the Mann–Whitney U-test was used.

Interval-scaled variables were tested for differences in averages between the groups using a *t*-test for independent samples, with adjustment of degrees of freedom for inhomogeneous variances. In addition, a binary regression analysis between CUR versus PUR was performed to identify risk factors associated with CUR. Following the univariate analysis, only variables with statistical significance were included for multivariate analysis. Given the distribution of each case (83 PUR vs. 29 CUR), a classification threshold was adjusted to reflect the prevalence of CUR of 25.9% and to ensure a balanced identification of the less frequent event. Odds ratios (ORs) were presented with 95% confidence intervals (95% CIs).

All statistical tests were performed at a 5% significance level. When *p* < 0.05 and <0.01, the results were considered to be statistically significant and highly significant, respectively. The statistical software package SPSS Statistics 26.0 (SPSS Inc., Chicago, IL, USA, version 26) was used for data analysis.

## 3. Results

The maternal characteristics of patients with uterine rupture are summarized in Table 1. In this population, 68 women (60%) were aged equal to or less than 35 years, while 44 women (40%) were older than 35 years, including nine women (8%) over 40. Most of the women (109 (97%)) were of average height. Regarding pre-pregnancy body mass index (BMI), 70 women (65%) had an average BMI (<25 kg/m^2^), 24 (22%) were overweight, and 14 (13%) were obese (BMI ≥ 30 kg/m^2^).

In terms of gravidity, five women (4%) were primigravida, forty-five (40%) were in their second pregnancy, and sixty-two (55%) were in their third-or-greater pregnancy. In seven women (6%), the de novo uterine rupture occurred during their first delivery, whereas 67 women (60%) had the rupture in their second birth and 38 (34%) during their third-or-greater birth. Although only five women were primigravida, seventeen women had a history of abortion, which accounts for the seven cases of rupture during a first delivery.

Eighty-four children (75%) were born at term, twenty-three (21%) were born at preterm, and five births (4%) were extremely preterm.

Analysis of prior cesarean deliveries revealed that most patients had undergone at least one previous C-section. In particular, 66 women (59%) had one prior C-section, 24 (21%) had undergone two, and 11 (10%) had experienced more than three C-sections. In contrast, 11 patients (10%) had never undergone a C-section. A total of 13 patients (12%) had a history of uterine rupture in previous pregnancies, while 14 (13%) had undergone prior uterine surgery other than C-sections. Regarding interpregnancy intervals, in 17 women (16%), the uterine rupture occurred during a delivery within one year following a previous birth. Gestational diabetes was present in fourteen patients (12%), whereas pregnancy-induced hypertension was observed in only one case (1%).

Labor induction was performed in twenty-four cases (21%), of which twelve (80%) received less than 5 mg of prostaglandin gel, and three (20%) received more than 5 mg. During labor, 44 women (43%) reported severe abdominal or scar-related pain. Premature rupture of membranes occurred in 26 cases (24%).

Regarding the time of delivery, 51 births (46%) occurred during regular working hours, while 61 deliveries (54%) took place outside of regular working hours.

Neonatal birth weight distribution showed that ten neonates (9%) were large for gestational age (LGA), ninety-four (84%) were appropriate for gestational age, and eight (7%) were small for gestational age (SGA). Considering body length and head circumference, 32 neonates (30%) were classified as LGA, and about 15% as SGA (15 for body length, 19 for head circumference).

Regarding the mode of C-section, secondary cesarean deliveries were nearly twice as common as primary C-sections (63 (57%) vs. 33 (30%) births). Emergency C-sections were performed in 15 cases (13%). The duration of hospitalization varied, with 94 women (85%) staying for three to five days and 13 (12%) remaining hospitalized for more than five days. Postpartum hemorrhage exceeding one liter occurred in seventeen women (16%), requiring blood transfusion in eleven cases (10%) and intensive care in nine (8%). Emergency hysterectomy was necessary in three cases (2.7%).

Neonatal APGAR scores improved over time. Analysis of umbilical artery pH revealed that ninety neonates (82.4%) had a normal pH (≥7.2), while twelve (11%) exhibited mild acidosis (≤7.1 and >7.2), three (2.7%) had moderate acidosis (≤7.0 and >7.1), and four (3.7%) experienced severe acidosis (<7.0). Neonatal hemoglobin levels ranged between 14 and 24 g/dL in 80 cases (84%), while 15 cases (16%) had reduced levels. Three neonates needed resuscitation. Transfer to NICU was necessary for 28 neonates (25%).

Next, we investigated the impact of type of uterine rupture on maternal and neonatal outcomes. The risks of prolonged hospitalization beyond five days (CUR, *n* = 6, 20.7%; PUR, *n* = 5, 6.0%, *p* = 0.003), blood loss exceeding one liter (CUR, *n* = 7, 24.1%; PUR, *n* = 6, 7.2%, *p* < 0.001), need for maternal transfusion (CUR, *n* = 7, 24.1%; PUR, *n* = 4, 4.8%, *p* = 0.003), and transfer to ICU (CUR, *n* = 6, 20.7%; PUR, *n* = 3, 3.6%, *p* = 0.004) were all significantly higher in cases of CUR compared to PUR. The incidence of postpartum emergency hysterectomy was low for both types of rupture (CUR, *n* = 2, 6.9%; PUR, *n* = 1, 1.2%, *p* = 0.11) and no maternal death was observed.

For neonatal outcomes, the risks of severe acidosis (CUR, *n* = 6, 20.7%; PUR, *n* = 0, 0%, *p* < 0.001), an APGAR score below 8 at five minutes (CUR, *n* = 11, 37.9%; PUR, *n* = 4, 4.8%, *p* < 0.001), a need for transfer to NICU (CUR, *n* = 13, 44.8%; PUR, *n* = 15, 18.1%, *p* = 0.004), and for resuscitation (CUR, *n* = 3, 10.3%; PUR, *n* = 0, 0%, *p* = 0.016) were all significantly higher in cases of CUR compared to PUR. One case of neonatal death, likely due to perinatal causes, was reported (Table 2).

The results of univariate and multivariate binary logistic regression analysis are shown in Table 3. Factors associated with an increased risk of CUR included pathological CTG (OR = 1.9, 95% CI: 0.99–7.14, *p* = 0.05), scar pain (OR = 2.63, 95% CI: 1.10–6.25, *p* = 0.03), no previous C-section (OR = 4.3, 95% CI: 1.23–16.6, *p* = 0.02), previous vaginal birth (OR = 7.14, 95% CI: 0.025–20, *p* < 0.001), and lack of uterine contractions (OR = 7, 95% CI: 1.21–40.46, *p* = 0.030) (Table 4). Patients with one previous C-section had a 4.94 times (OR = 4.94, 95% CI: 1.38–17.67, *p* = 0.014) higher risk of CUR, whereas patients with more than two C-sections had a 0.66 times (OR = 0.66, 95% CI: 0.13–3.22, *p* = 0.61) lower risk of CUR. After multivariate analysis, the independent risk factors that remained significantly associated with CUR were abdominal/scar pain (OR = 4, 95% CI: 1.39–11.11, *p* = 0.01) and previous vaginal birth (OR = 4, 95% CI: 1.00–16.67, *p* = 0.049) (Table 3). De novo uterine rupture without any previous uterine surgery occurred in five cases (4.5%), and of which resulted in CUR. There was a non-significant trend toward increased CUR risk with delivery outside working hours (OR = 2.11, 95% CI: 0.86–5.18, *p* = 0.1) and with labor induction (OR = 2.27, 95% CI: 0.88–5.88, *p* = 0.09). The amount of prostaglandins used did not significantly affect the risk of CUR (3 mg, OR 1.17, 95% CI: 0.29–4.76, *p* = 0.83; 4 mg, OR = 2.12, 95% CI: 0.34–14.28, *p* = 0.422). TOLAC with or without labor induction did not increase the risk of CUR. Neonatal characteristics including birth length, birth weight, head circumference, and fetal malposition, as well as maternal characteristics such as age, BMI, and height, medical history had no significant impact on the risk of CUR. No association with increased risk of CUR was found in cases of prolonged/obstructed labor (OR = 1.25, 95% CI: 0.23–6.67, *p* = 0.80), birth in two consecutive years (OR = 0.51, 95% CI: 0.11–2.30, *p* = 0.39), premature membrane rupture (OR = 1.78, 95% CI: 0.69–4.55, *p* = 0.23), or myoma enucleation (OR = 0.6, 95% CI: 0.07–4.35, *p* = 0.65).

Finally, we aimed to assess whether there were significant differences in measurable pre- and antepartum factors between maternal and neonatal high-risk ruptures and CUR. Maternal high-risk rupture was characterized by blood loss exceeding one liter eventually necessitating transfusion and admission to ICU. No significant associations were identified between maternal high-risk rupture and CUR. In contrast, neonatal high-risk ruptures (defined by a need for NICU admission) were significantly associated with differences in parity, gestational age, birth weight, and the number of prior C-sections compared to CUR (Table 4).

## 4. Discussion

Identification of patients at risk of uterine rupture remains a significant challenge in obstetric care. Consistent with previous studies [19,20], our analysis confirmed that cases of CUR are associated with serious maternal and neonatal outcomes, highlighting the critical importance of accurate recognition of such high-risk uterine ruptures.

Labor induction with oxytocin and/or prostaglandins has been identified as a significant risk factor for uterine rupture [11,19]. In our study, labor induction was conducted in fifteen of eighty-three women with PUR (18%), and in nine of twenty-nine women with CUR (45%), representing more than twice the proportion observed in PUR. The amount of prostaglandins used did not significantly affect the risk of CUR (3 mg, OR 1.17, 95% CI: 0.29–4.76, *p* = 0.83; 4 mg, OR = 2.12, 95% CI: 0.34–14.28, *p* = 0.422). Consistent with previous studies, our results showed a trend indicating that the risk of CUR increased in cases of labor induction (OR = 2.27, 95% CI: 0.88–5.88, *p* = 0.09), thus highlighting the importance of careful patient selection for labor induction.

Al-Zirqi et al. reported that delivery times after midnight increased the risk of intrapartum and infant death by 4.3 times compared to delivery times between 08:00 and 15:00 [21]. In our study, 61 births (54%) happened outside usual working hours, and CUR occurred more often during these times (OR = 2.1, 95% CI: 0.86–5.18, *p* = 0.1). In further analysis, the risk of CUR at times other than between 08:00 and 17:00 was found to be 2.33 times (OR = 2.33, 95% CI: 0.99–5.56, *p* = 0.053) higher. During nighttime, or outside regular working hours, fewer healthcare professionals are available, and fatigue may be more prevalent, potentially resulting in delayed responses to complications. Furthermore, studies suggest that the onset and progression of labor may be influenced by circadian rhythms, potentially affecting the timing and likelihood of obstetric complications [22].

Women with previous vaginal birth had a significantly higher risk of CUR compared to PUR (OR = 7.14, 95% CI: 0.025–20, *p* < 0.0001). Moreover, previous vaginal birth remained an independent risk factor for CUR even after multivariate analysis (OR = 4, 95% CI: 1.00–16.67, *p* = 0.049), contrary to general expectation. One possible explanation for this finding is that a prior C-section is a well-established risk factor for uterine rupture, leading to a lower threshold for recommending a repeat cesarean delivery. In contrast, a history of vaginal birth is often considered a favorable predictor for successful vaginal delivery, making women with previous vaginal birth more likely to be motivated to attempt TOLAC. Navum-Yerushalmy et al. demonstrated that a prior vaginal birth serves as a protective factor against uterine rupture in patients attempting TOLAC [23]. Similarly, Atia et al. reported that a previous VBAC is predictive for both TOLAC success and increased risk of uterine rupture [24]. However, the number of prior vaginal deliveries does not appear to improve predictive accuracy. In the present study, 18 cases with a history of previous vaginal birth were identified. Among these, six women experienced PUR and twelve experienced CUR. The number of previous vaginal births was either one (n = 14), two (n = 3), or three (n = 1). The patient with three prior vaginal births experienced CUR, as did one of the three patients with two prior vaginal births. The remaining 10 CUR cases occurred in women with one previous vaginal birth. Due to the retrospective nature of the study, the chronological order of vaginal births and C-sections could not be definitively determined. Another potential explanation is that uterine dehiscences, which may occur during labor, are often detected and repaired during C-sections. However, in cases of vaginal delivery, an existing uterine lesion may go unnoticed if it remains asymptomatic. In a subsequent pregnancy, such an undiagnosed lesion could expand, increasing the risk of complications, including CUR. Because routine postpartum assessment for a uterine dehiscence is not a feasible clinical option, there are no studies to support this theory. Consistent with our findings, Dimitrova et al. reported that women in a TOLAC group with higher numbers of previous vaginal births had an increased risk of CUR compared to PUR [20]. This finding confirms that a history of vaginal births does not eliminate the risk of uterine rupture and also that, when rupture does occur, it is more likely to result in CUR.

The risk of CUR was significantly higher for women without previous C-section (OR = 4.3, 95% CI: 1.23–16.6, *p* = 0.02). One previous C-section further increased the risk of CUR by 4.94 times (OR = 4.94, 95% CI: 1.38–17.67, *p*  = 0.014), whereas patients with more than two C-sections had a lower risk of CUR (OR = 0.66, 95% CI: 0.13–3.22, *p* = 0.61).

Among the eleven women without a prior C-section, ten experienced CUR, with only one case resulting in PUR. However, five of these patients had undergone myoma enucleation. One patient had a salpingectomy, a similar case of which was reported for uterine rupture in nulliparous women [25]. Another patient had FIGO Stage IIIC ovarian cancer, characterized by abdominal peritoneal carcinomatosis, which may have contributed to childbirth complications, likely by impairing cervicouterine dilatation. These findings suggest that even in the absence of direct uterine manipulation, pelvic pathology may influence uterine integrity. Studies showed that ruptures of unscarred uteri were more frequently classified as CUR, leading to worse maternal and neonatal outcomes [26,27]. The risk factors for spontaneous CUR in unscarred uteri remain insufficiently examined. Thisted et al. examined 20 cases of CUR in unscarred uteri and identified associations with multiparity, epidural analgesia, and oxytocin augmentation [28]. Ofir et al. analyzed 53 CUR cases, including 26 patients with a scarred uterus and 27 patients without a uterine scar, and found that cervical involvement was more common in unscarred uteri. Factors such as higher birth order, uterine tachysystole, and fetal macrosomia were more frequent in the unscarred uteri without reaching statistical significance. Maternal and perinatal morbidity rates were comparable between groups [29]. In our study, four patients experienced de novo CUR. These four patients varied in age, BMI, gravidity, parity, and gestational age. Three of the four underwent labor induction with prostaglandin tablets. The risk of CUR in unscarred uteri was 2.13 times higher than that of PUR; however, this difference did not reach statistical significance (*p* = 0.42). Another factor associated with an increased risk of CUR was a uterus without contraction (OR = 7, 95% CI: 1.21–40.56, *p* = 0.30), with seven such cases reported, each exhibiting different obstetrical characteristics that resulted in CUR. One possible explanation is that these high-risk ruptures, occurring in the absence of typical risk factors, may require significant intrauterine or intra-abdominal pressure, which could directly contribute to the development of CUR. However, due to the limited number of cases, further analysis was not feasible.

In our study, 35 of 66 women (53%) with one previous C-section attempted TOLAC, accounting for 85% of all TOLAC attempts. Among these patients, 10 experienced CUR, representing 34% of all CUR cases. A history of more than two C-sections was not identified as a risk factor for CUR, as a repeat C-section is the standard mode of delivery after more than two C-sections. Mao et al. reported that the risk of uterine rupture and maternal mortality is increased after two C-sections compared to a repeat C-section [30]. Furthermore, Landon et al. observed significantly higher rates of emergency hysterectomy and blood transfusion in women with multiple prior C-sections compared to those with one previous C-section [19]. Those findings were not presented in our study. This may be attributed to a well-standardized management system in a specialized prenatal center for women with risk pregnancies, including multiple C-sections [31].

A significant risk factor that remained after multivariate analysis was persistent scar/abdominal pain (univariate: OR = 2.63, 95% CI: 1.10–6.25, *p* = 0.03; multi-variate: OR = 4, *p* = 0.01). Sgayer et al. conducted a review of the literature on spontaneous CUR and found that abdominal pain was the most common clinical symptom, being present in 77.4% of cases [5]. Although their study did not directly compare cases of CUR to other complications, this finding suggests that severe abdominal pain is a significant indicator of more complicated birth outcomes. Additionally, a study by Savukyne et al. identified acute abdominal pain during labor as the most frequently reported symptom of uterine rupture [8]. It is important to note that uterine rupture can be preceded or accompanied by various changes in uterine contractility, including hyperstimulation, reduced contractions, or alterations in baseline uterine tone; however, no consistent pattern has been observed, making these changes non-specific [32]. Furthermore, women with epidural anesthesia experience less pain, making it more difficult to recognize the rupture [28]. For clinicians, it is crucial to take abdominal pain seriously, particularly when additional risk factors are present, in order to make appropriate decisions regarding the mode of delivery.

We also compared cases of CUR (n = 29) involving maternal (n = 17) or neonatal high-risk rupture (n = 28). Pre- and antepartum factors, including maternal age, gravidity, parity, maternal physical characteristics such as height, BMI, weight gain during pregnancy, gestational age, mode of previous delivery, intervals between previous births, gestational diabetes, and induction of labor, as well as fetal characteristics such as birth weight, length, head circumference, and fetal position, were compared between the groups. No significant differences were found between maternal high-risk rupture and CUR. However, significant differences were noted between neonatal high-risk rupture and CUR, particularly regarding gestational age, which subsequently influenced birth weight. Additionally, a higher number of previous C-sections, especially in multiparous women, was associated with neonatal high-risk rupture. This may be attributed to prolonged labor due to scar tissue and adhesions from prior surgeries, which could interfere with normal uterine function and cause fetal distress or hypoxia.

A substantial limitation of the present study arises from its retrospective nature because of inconsistencies in the documentation of uterine rupture, particularly in distinguishing between CUR and PUR. Due to the rarity of uterine rupture, it took 11 years to collect over 100 cases. Furthermore, the limited sample size restricted the feasibility of subgroup analyses of interest. Additionally, the study analyzed only women with a history of C-section, raising the risk that asymptomatic uterine ruptures occurring in vaginal deliveries might have gone undetected. Another limitation of the study is the lack of post-discharge follow-up to assess maternal and neonatal complications, which prevents the identification of potential long-term adverse outcomes. Because the study was conducted at a level 1 perinatal center, where comprehensive antepartum and peripartum obstetric management was ensured, the findings may not be generalized to all healthcare facilities. Finally, the lack of a control group of women without uterine rupture limits the ability to compare risk factors and to accurately assess the relative risks associated with uterine rupture. The absence of a control group may restrict the generalizability of the result. Despite its limitations, the present study addresses a rare but life-threatening obstetric complication through a retrospective analysis of over 100 cases from a single center. The relatively large number of cases of such an uncommon event strengthens the clinical relevance of the findings. These meaningful clinical observations may contribute valuable insights to the limited literature on uterine rupture and help inform future research and clinical practice.

## 5. Conclusions

In conclusion, our study assessed risk factors and outcomes associated with different types of uterine rupture and sought to predict clinical high- and low-risk uterine ruptures according to prepartum factors. We identified vaginal birth and abdominal pain as key risk factors for complete uterine rupture, which leads to severe maternal and neonatal outcomes. A history of previous vaginal births does not entirely rule out the risk of uterine rupture. Abdominal pain should be taken seriously, especially when other risk factors are present. The study supports a stratified risk approach by distinguishing between complete and partial ruptures, as well as offering practical guidance for patient counseling, labor management, and TOLAC selection. To verify clinically the high-risk uterine ruptures reported here, further studies with larger sample sizes should be conducted.

## Figures and Tables

**Table 1:** Summary of maternal and neonatal characteristics and outcomes.

Maternal Characteristics				
Age (years)	<35	35–40		≥40
	68 (60%)	44 (40%)		9 (8%)
Height (cm)	<153	153–180		≥180
	3 (2.6%)	109 (97%)		1 (0.9%)
BMI (kg/m^2^)	<25	25–30		≥30
	70 (65%)	24 (22%)		14 (13%)
**Obstetrical characteristics**				
Gravidity	Primigravida	Bigravida		Multigravida
	5 (4%)	45 (40%)		62 (55%)
Parity	Primipara	Bipara		Multipara
	7 (6%)	67 (60%)		38 (34%)
Gestational age (weeks + days)	Extremely preterm (22 + 0–27 + 6)	Preterm (28 + 0–36 + 6)		Term >37 + 0
	5 (4%)	23 (21%)		84 (75%)
Previous C-section ^#^	None	1		≥2
	11 (10%)	66 (59%)		35 (31%)
Previous vaginal birth ^#^	None	1	2	3
	94 (83.9%)	14 (12.5%)	3 (2.7%)	1 (0.9%)
**Pregnancy risk factors**				
	Yes		No	
Previous uterine rupture ^#^	13 (12%)		99 (88%)	
Birth in two consecutive years ^#^	7 (6%)		105 (94%)	
Previous operation on uterus other than C-section ^#^	14 (13%)		98 (87%)	
TOLAC ^#^	41 (37%)		71 (63%)	
Gestational diabetes	14 (12%)		98 (88%)	
Hypertonia	1 (1%)		111 (99%)	
Labor induction ^#^	24 (21%)		88 (79%)	
Scar/abdominal pain ^#^	44 (43%)		59 (57%)	
Premature membrane rupture	26 (24%)		82 (76%)	
Birth during usual working hours ^#^	51 (46%)		61 (54%)	
**Neonatal characteristics**				
	SGA (<10. percentile)	Norm (≥10. < 90. percentile)		LGA (≥90. percentile)
Weight	8 (7%)	94 (84%)		10 (9%)
Length	15 (14%)	63 (57%)		32 (29%)
Head circumference	19 (17%)	58 (53%)		33 (30%)
**Maternal outcome**				
Type of C-section ^#^	Primary	Secondary		Emergency
	33 (30%)	63 (57%)		15 (13%)
Hospitalization (days)	<3	3–5		≥5
	4 (3%)	94 (85%)		12 (12%)
Blood loss	<1000 mL		≥1000 mL	
	88 (84%)		17 (16%)	
Need of transfusion	Yes		No	
	11 (10%)		101 (90%)	
Transfer to ICU	Yes		No	
	9 (8%)		103 (92%)	
Emergency hysterectomy	Yes		No	
	3 (2.7%)		109 (97.3%)	
**Neonatal outcome**				
APGAR	0–4	5–8		9–10
At 1 min	13 (12%)	31 (28%)		67 (69%)
At 5 min	4 (4%)	29 (26%)		78 (70%)
At 10 min	1 (1%)	13 (12%)		97 (87%)
pH of umbilical artery	<7	7.0–7.09	7.1–7.19	≥7.2
	4 (3.6%)	3 (2.7%)	12 (11%)	90 (82.7%)
Base excess	<−10	−10–−5	−5–0	≥0
	10 (9%)	12 (11%)	79 (72%)	9 (8%)
Hemoglobin after birth (g/dL)	≥14		<14	
	80 (84%)		15 (16%)	
Need of resuscitation	Yes		No	
	3 (3%)		107 (97%)	
Transfer to NICU	Yes		No	
	28 (25%)		82 (75%)	
Death	Yes		No	
	1 (1%)		111 (99%)	

^#^ Factors, which were further analyzed in the univariate and multivariate binary logistic regression analysis for CUR (Table 3).

**Table 2:** Comparison of maternal and neonatal outcomes between CUR and PUR.

	PUR (n = 83)	CUR (n = 29)	*p*-Value
**Maternal outcome**			
Emergency hysterectomy	1 (1.2%)	2 (6.9%)	0.11
Hospitalization > 5 days	5 (6.0%)	6 (20.7%)	0.003 *
Transfusion	4 (4.8%)	7 (24.1%)	0.003 *
Blood loss > 1000 mL	6 (7.2%)	11 (37.9%)	<0.001 *
Transfer to ICU	3 (3.6%)	6 (20.7%)	0.004 *
Death	0 (0%)	0 (0%)	
**Neonatal outcome**			
Severe acidosis (pH ≤ 7.0)	0 (0%)	6 (20.7%)	<0.001 *
APGAR score below 8 at 5 min	4 (4.8%)	11 (37.9%)	<0.001 *
Transfer to NICU	15 (18.1%)	13 (44.8%)	0.004 *
Hemoglobin lower than 14 g/dL	12 (14.5%)	3 (10.3%)	0.576
Need for resuscitation	0 (0%)	3 (10.3%)	0.016 *
Death	0 (0%)	1 (3.4%)	0.259

* Statistically significant.

**Table 3:** Univariate and multivariate binary logistic regression analysis for CUR.

	Univariate OR (95% CI)	*p*-Value	Multivariate Adjusted OR (95% CI)	*p*-Value
Pathological CTG	1.9 (0.99–7.14)	0.05 *	1.57 (0.53–5.88)	0.35
Prolonged/obstructed labor	1.25 (0.23–6.67)	0.80		
Birth outside of regular working hours	2.11 (0.86–5.18)	0.10		
Primary vs. secondary C-section	0.33 (0.1–1.03)	0.06		
Scar/abdominal pain	2.63 (1.10–6.25)	0.03 *	4 (1.39–11.11)	0.01 *
Birth in two consecutive years	0.51 (0.11–2.30)	0.39		
Premature membrane rupture	1.78 (0.69–4.55)	0.23		
Previous uterine rupture	0.25 (0.03–2.04)	0.20		
Myoma enucleation	0.6 (0.07–5.37)	0.65		
Labor induction	2.27 (0.88–5.88)	0.09		
TOLAC	1.79 (0.75–4.35)	0.19		
TOLAC with labor induction	1.47 (0.36–5.95)	0.59		
Unscarred uteri	2.13 (0.34–13.44)	0.42		
No previous C-section	4.3 (1.23–16.6)	0.02 *	2.7 (0.61–11.11)	0.19
One previous C-section	4.94 (1.38–17.67)	0.014 *	3.19 (0.77–13.28)	0.11
More than two previous C-sections	0.66 (0.13–3.22)	0.61		
Previous vaginal birth	7.14 (0.025–20)	<0.001 *	4 (1.00–16.67)	0.049 *
No uterine contraction	7 (1.21–40.56)	0.030 *	2.06 (0.23–18.71)	0.52

* Statistically significant.

**Table 4:** Comparison of maternal and neonatal high-risk uterine ruptures with CUR.

	Maternal High-Risk Rupture (n = 17)	CUR (n = 29)	*p*-Value	Neonatal High-Risk Rupture (n = 28)	CUR (n = 29)	*p*-Value
Age (years; average ± SD)	35.94 ± 4.8	35.38 ± 4.2	0.34	33.54 ± 4.1	35.38 ± 4.2	0.051
Gravidity (average ± SD)	2.47 ± 0.9	2.48 ± 1.1	0.49	3.25 ± 1.5	2.48 ± 1.1	0.02
Parity (average ± SD)	2.0 ± 0.6	2.07 ± 0.9	0.40	2.54 ±1.1	2.07 ± 0.9	0.049 *
Height (cm; average ± SD)	167.18 ± 6.4	167 ± 5.6	0.46	165.68 ± 5.9	167 ± 5.6	0.199
BMI (kg/m^2^; average ± SD)	23.46 ± 3.3	23.3 ± 4.0	0.45	24.01 ± 4.7	23.3 ± 4.0	0.27
Weight gain during pregnancy (kg; average ± SD)	10.35 ± 7.9	10.25 ± 6.6	0.48	12.82 ± 6.3	10.25 ± 6.6	0.06
Gestational age (weeks + days; average ± SD)	36.65 ± 4.3	36.55 ± 4.3	0.47	33.89 ± 4.2	36.55 ± 4.3	0.012 *
Birth weight (g; average ± SD)	2942.41 ± 1048.9	2963.1 ± 1016.5	0.47	2487.14 ± 896.9	2963.1 ± 1016.5	0.036 *
Birth length (cm; average ± SD)	50.12 ± 6.4	49.3 ± 6.7	0.35	46.35 ± 6.3	49.3 ± 6.7	0.051
Fetal head circumference (cm; average ± SD)	33.81 ± 3.8	33.39 ± 3.9	0.45	32.09 ±3.7	33.39 ± 3.9	0.11
Number of previous C-sections (average ± SD)	0.82 ± 0.71	0.76 ± 0.68	0.38	1.29 ± 1.03	0.76 ± 0.68	0.014 *
Fetal malposition	2 (11.8%)	4 (13.8%)	0.84	5 (17.9%)	4 (13.8%)	0.67
Birth in two consecutive years	2 (11.8%)	3 (10.3%)	0.88	4 (14.3%)	3 (10.3%)	0.65
Labor induction	6 (35.3%)	9 (31.0%)	0.77	4 (14.3%)	9 (31.0%)	0.13
Gestational diabetes	2 (11.8%)	2 (6.9%)	0.57	3 (10.7%)	2 (6.9%)	0.61

* Statistically significant.

## Data Availability

Further inquiries about data can be directed to the corresponding author.
